# A Benign Ulcerating Gastric Mass Presenting as Acute Gastrointestinal Hemorrhage

**DOI:** 10.7759/cureus.15954

**Published:** 2021-06-27

**Authors:** Yasmeen Obeidat, Joseph Simmons, Saba AlTarawneh, Saroj Sigdel, Wesam Frandah, Elizabeth Saunders

**Affiliations:** 1 Internal Medicine, Marshall University Joan C. Edwards School of Medicine, Huntington, USA; 2 Pathology, Marshall University Joan C. Edwards School of Medicine, Huntington, USA; 3 Gastroenterology and Hepatology, Marshall University Joan C. Edwards School of Medicine, Huntington, USA

**Keywords:** bleeding lipoma, rare gastric tumor, upper gastro-intestinal bleed, benign tumors, gastrointestinal symptoms

## Abstract

Gastrointestinal lipomas are rare, often colonic tumors. The stomach is an unusual site of involvement of lipomas, accounting for less than 5% of all gastrointestinal lipomas and less than 3% of all benign gastric neoplasms. They are usually asymptomatic, and symptoms develop as the tumor grows. Gastric lipomas can present with massive bleeding from an ulcerating tumor and can be life-threatening if left untreated. We present a case of an ulcerating gastric lipoma that presented as an acute upper gastrointestinal hemorrhage. The patient was treated with Billroth II procedure and final pathology showed an ulcerating submucosal lipoma. The diagnosis of gastric lipoma is often suspected incidentally on imaging, then confirmed via biopsy. Definitive treatment of large lesions typically requires surgery, however, newer endoscopic techniques are being utilized for resection of these benign tumors.

## Introduction

Lipomas are benign tumors that form from mature adipose tissue [1]. Gastrointestinal lipomas are rare and, if present, are usually found in the colon [1-3]. The stomach is an unusual site of involvement of lipomas, accounting for less than 5% of all gastrointestinal lipomas and less than 3% of all benign gastric neoplasms [1-4]. When found, 75% of gastric lipomas are located in the antrum and are typically submucosal in origin but can be subserosal in rare cases [1,4-6]. They are usually asymptomatic and discovered incidentally, however bleeding, dyspeptic symptoms, intussusception, and gastric outlet obstruction can occur, most commonly when the lipoma is greater than 2.0 cm in size [1,4-8]. Malignant transformation of these gastric lipomas is rare, however, large size and symptoms of outlet obstruction can necessitate intervention [9]. There are a limited number of case reports of gastric lipomas reported in the literature, and even fewer report gastrointestinal hemorrhage as the presenting symptom. We present a case of gastrointestinal hemorrhage secondary to an ulcerating gastric lipoma.

## Case presentation

**Figure 1:**
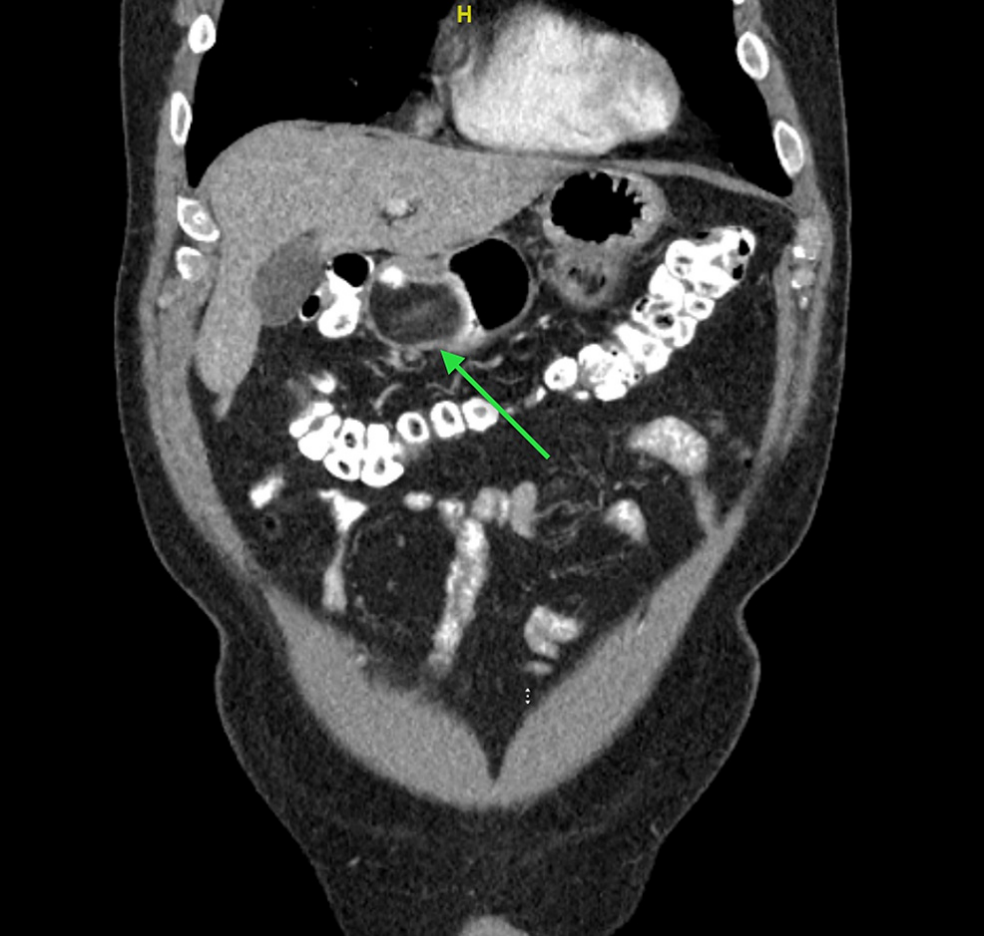
A fat-attenuating mass at the gastric antrum protruding through the pylorus and into the first portion of the duodenum.

**Figure 2:**
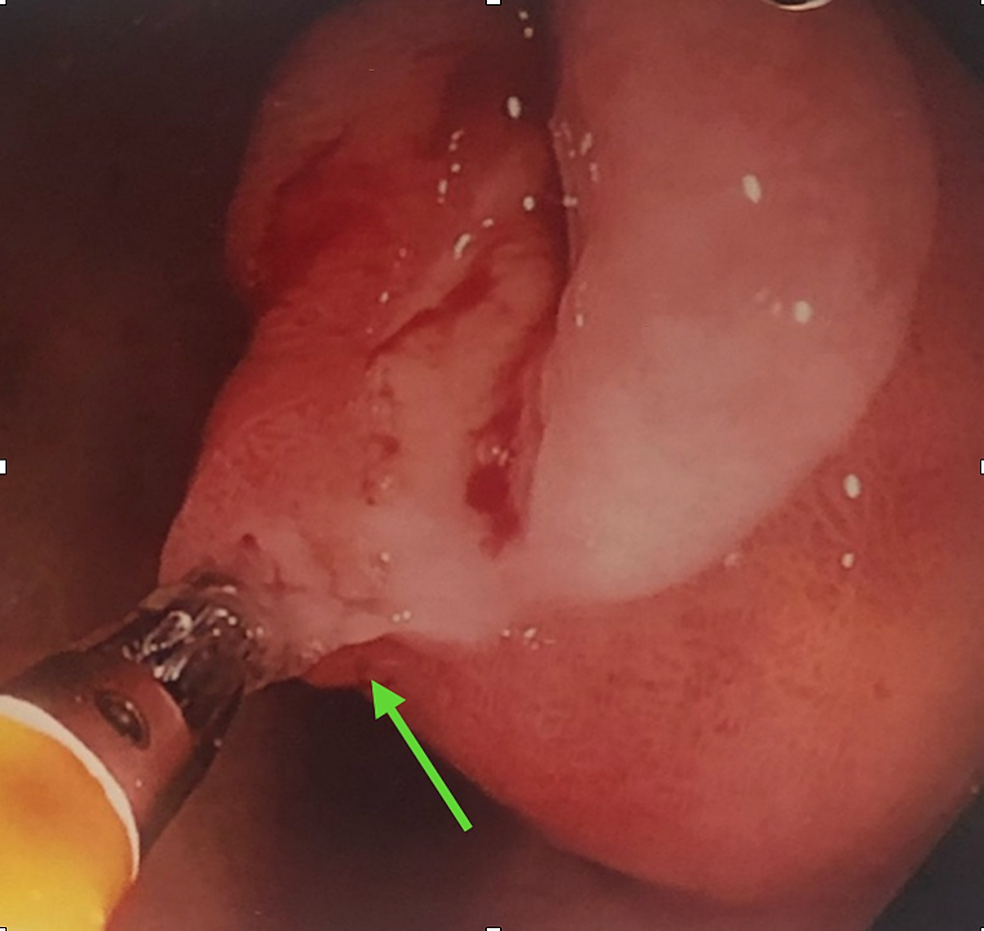
Endoscopic view of lipoma showing “tenting” sign and bleeding secondary to ulceration.

**Figure 3:**
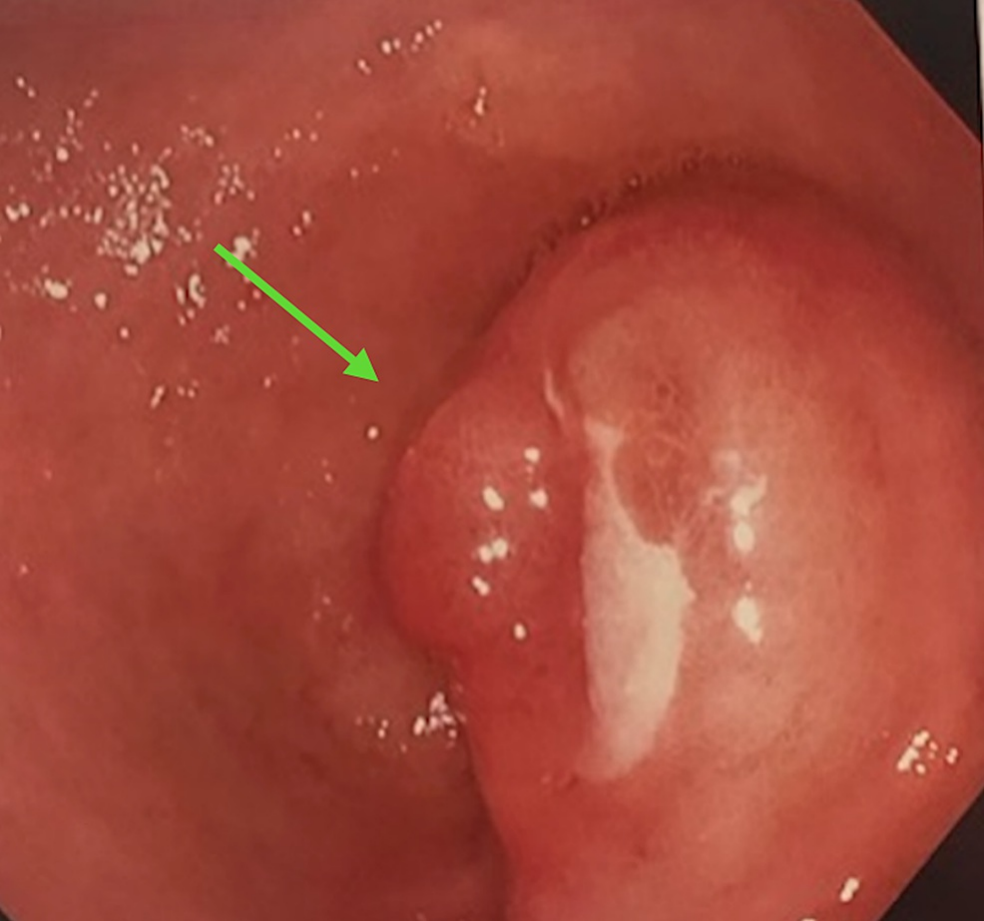
Endoscopic view of the lipoma causing outlet obstruction at the pylorus.

A 65-year-old male with a known past medical history of hypertension, gastroesophageal reflux disease, and hyperlipidemia presented with complaints of intermittent, non-localized abdominal discomfort associated with early satiety, decreased appetite, and shortness of breath on exertion for six months duration. His symptoms acutely worsened two weeks prior to presentation and were associated with black, tarry stools of four days duration. Further history revealed alcohol consumption of 1-2 beers per day and a previous 60 pack-year cigarette smoking history. He denied ever having an esophagogastroduodenoscopy (EGD) but did have a colonoscopy 12 years prior to presentation which revealed diverticulosis and a single polyp that was successfully removed. Vital signs were stable and physical examination revealed no abdominal tenderness, abdominal distension, or conjunctival pallor. Laboratory results revealed a hemoglobin level of 9.7 mg/dL (decreased from his baseline value of 15 mg/dL), hematocrit level of 28.7%, and a mean corpuscular volume (MCV) of 95.5 fL. The remainder of his laboratory values including platelet count, white blood cell count, serum creatinine, blood urea nitrogen (BUN), potassium, sodium, and bicarbonate were within normal limits. He was started on pantoprazole twice daily, and intravenous fluid resuscitation was initiated. The patient underwent computed tomography (CT) scan of his abdomen and pelvis with intravenous contrast that revealed evidence of a 5 cm fat-attenuating mass at the gastric antrum protruding through the pylorus and into the first portion of the duodenum, suggestive of lipoma. Hemoglobin level dropped to 8.2 mg/dL during his admission. The patient underwent an EGD which revealed a large, ulcerated mass in the antrum of the stomach with central necrosis that appeared like an atypical ulcer (Figures 1-3). Given the appearance of the mass, a neoplastic process such as a gastrointestinal stromal tumor was suspected and biopsies were obtained. 

**Figure 4:**
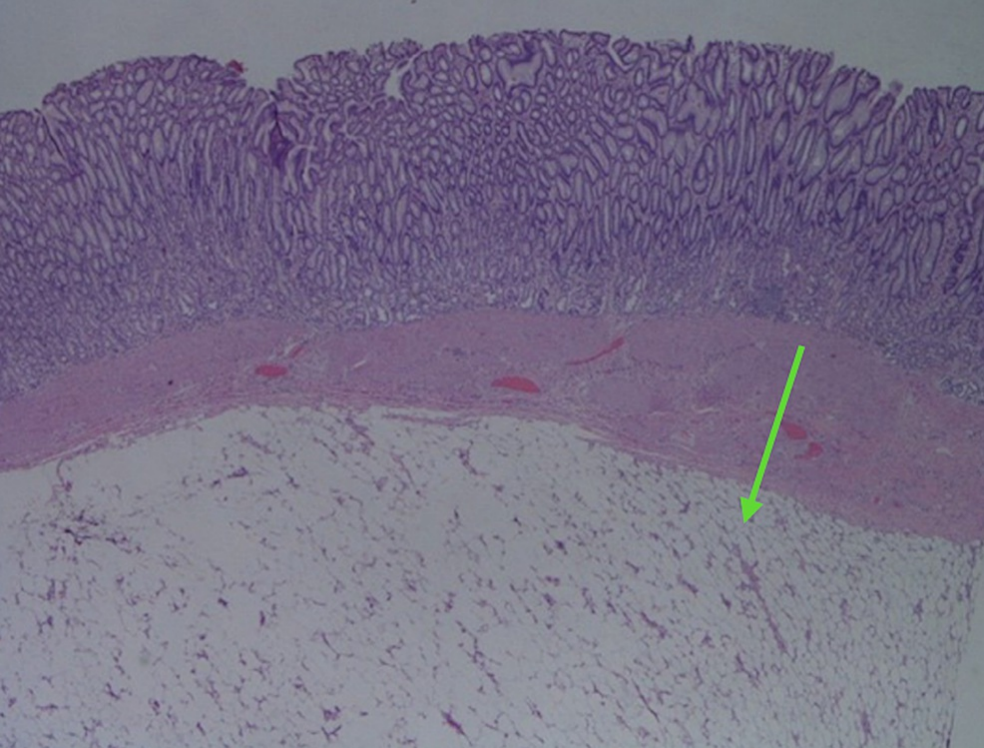
Gastric mucosa with well circumscribed submucosal adipose tissue consistent with lipoma (hematoxylin-eosin, original magnifications x40).

**Figure 5:**
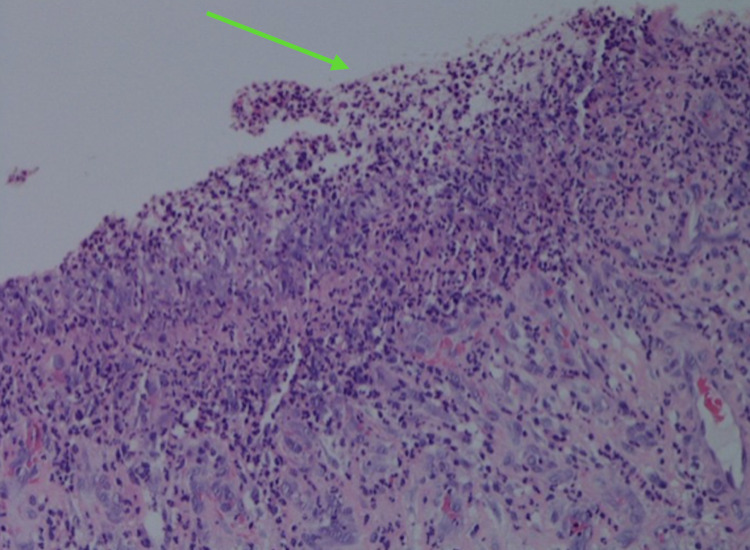
Gastric mucosa with evidence of ulceration, marked acute inflammation, and granulation tissue (hematoxylin-eosin, original magnifications x200).

**Figure 6:**
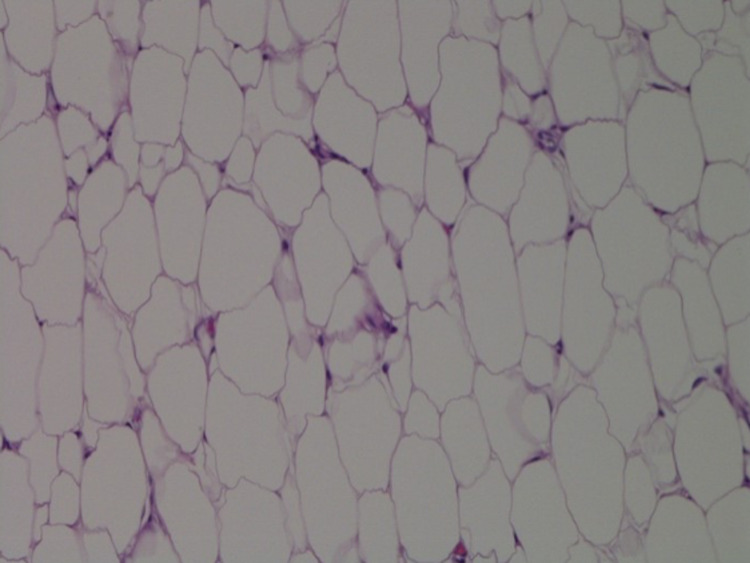
Lipoma showing mature adipose tissue with no significant cytologic atypia and no mitotic figures are identified (hematoxylin-eosin, original magnifications x400).

Biopsy results showed benign gastric mucosa with hyperplastic changes consistent with a hyperplastic polyp, and no malignant or dysplastic process was identified. Given the symptomatic nature of the mass and ongoing blood loss, the patient underwent an exploratory laparotomy. This revealed a gastric antral mass 5-6 cm in diameter with three sites of ulcerations without any evidence of lymph node or liver metastasis. A distal gastrectomy with gastrojejunostomy (Billroth II) procedure was performed. Surgical pathology revealed an ulcerated submucosal lipoma with six benign lymph nodes (Figures 4-6). Immunohistochemical staining was negative for Helicobacter pylori (H. pylori). The procedure was well-tolerated, and the patient had significant improvement of his symptoms. He was discharged home with close outpatient follow-up. 

## Discussion

Lipomas are tumors of mesenchymal origin that are slow-growing, benign, and surrounded by a thin fibrous capsule [2,6,7]. They can develop in any organ throughout the body, including the gastrointestinal tract. Gastrointestinal lipomas are most commonly found in the colon followed by the ileum and jejunum. The stomach is a less common site of occurrence for lipomas, accounting for only 5% of all lipomas of the gastrointestinal tract [3,4,6]. Other fatty tumors of the gastrointestinal tract include lipoblastomas (occurring in infants and children), hibernomas, atypical lipomatous tumors (low-grade sarcomas with little potential for metastasis), and liposarcomas [2,6].

Gastric lipomas arise from the submucosa in 95% of cases and are typically found in the gastric antrum (75%) [6,9]. Although definitive risk factors for the development of gastric lipomas have not been described, lipomas are generally observed in patients who are obese, diabetic, or in those who are greater than 45 years of age. These tumors can also be seen in patients who undergo a period of rapid weight gain; however, they do not decrease in size in patients who lose weight [6]. Gastric lipomas are usually asymptomatic and diagnosed incidentally. Symptoms develop when the tumor grows in size causing early satiety, dyspepsia, and gastric outlet obstruction. Hematemesis and/or melena can be present due to bleeding from an ulcerating tumor and can be life-threatening if left untreated [1,2,4-6,9]. Our patient developed significant gastrointestinal bleeding from multiple sites of the ulcerating tumor. On physical exam, patients can present with fullness in the epigastric area, palpation of a distinct mass, or an overall normal exam [2,6].

The diagnosis of gastric lipoma is often made incidentally when obtaining imaging for an unrelated issue or complaint. CT scan is specific for the diagnosis of lipomas, which are often described as well-circumscribed submucosal mass lesions with fat attenuation [3-5,7]. If linear strands of soft-tissue attenuation are present on imaging, ulceration and fibrovascular septa may be present which raises suspicion for liposarcoma [6]. MRI is another imaging modality that may be used to aid in the diagnosis of lipoma, though less commonly used. Lipomas typically have high signal intensity when viewing T1-weighted MRI images [6,10]. Although CT scan is specific in diagnosing lipomas, EGD is a useful diagnostic procedure for pre-operative diagnosis and further evaluation [2,6,9]. Evaluation of these masses with EGD often shows a soft, submucosal mass with a smooth surface that is yellow in color. Conversely, malignant lesions are often friable and have an ulcerated surface [6,10]. Upon evaluation via EGD, there are three signs which are suggestive of lipoma. These signs are: 1) Pillow sign - pressure from the biopsy forceps causes an indentation on the mass. 2) Tenting sign - the mucosa overlying the mass is easily moved with the biopsy forceps. 3) Naked fat sign - protruding fat through the mucosa after biopsies have been performed [1,2,6,9].

There are multiple modalities for the treatment of gastric lipomas. Asymptomatic lesions that are incidentally identified on imaging can be observed with no further treatment or evaluation [1,2,6]. Small-sized lesions (less than 2 cm) can be successfully excised endoscopically, but larger lesions often require surgical resection or a provider trained in advanced endoscopic procedures for a non-surgical approach [3,5,9,11]. Histopathologic evaluation of the removed tumor reveals mature fat cells with varying shapes and sizes which are usually larger than surrounding fat. These cells have regular nuclei, no hyperchromasia, and lack cytologic atypia. Any fatty tumor, both benign and malignant, will stain positively for vimentin and S-100 protein [6].

## Conclusions

Gastric lipomas are rare, benign, typically submucosal tumors that predominately occur in the gastric antrum. They are often found incidentally but can also present with symptoms of gastric outlet obstruction or gastrointestinal hemorrhage, such as in our case. Diagnosis of these lesions can be done based on typical CT findings alone, but further investigation with endoscopic or surgical biopsy may be necessary. Treatment options vary depending on the size of the lipoma and the availability of advanced endoscopic techniques. Small (< 2 cm), asymptomatic lesions can be observed or removed endoscopically. Larger (> 2 cm) lesions typically require surgical excision by a provider trained in advanced endoscopic procedures. Histopathologic evaluation of these tumors reveals mature fat cells with varying shapes and sizes, which are typically larger than the surrounding fat. All fatty tumors, both benign and malignant, will stain positively for vimentin and S-100 protein. Prognosis varies depending on the size of the lesions and symptoms. All bleeding lipomas should be removed to prevent potentially life-threatening hemorrhage.
